# Surveillance of HIV assisted partner services using routine health information systems in Kenya

**DOI:** 10.1186/s12911-016-0337-9

**Published:** 2016-07-20

**Authors:** Peter Cherutich, Matthew Golden, Bourke Betz, Beatrice Wamuti, Anne Ng’ang’a, Peter Maingi, Paul Macharia, Betsy Sambai, Felix Abuna, David Bukusi, Mathew Dunbar, Carey Farquhar

**Affiliations:** Ministry of Health, Nairobi, Kenya; University of Washington, Seattle, USA; Kenyatta National Hospital, Nairobi, Kenya; National AIDS/STI Control Programme (NASCOP), Kenyatta Hospital Grounds, off Hospital Road, Nairobi, Kenya

**Keywords:** Surveillance, Assisted partner services, HIV, Health information, Open data kit, Kenya

## Abstract

**Background:**

The utilization of routine health information systems (HIS) for surveillance of assisted partner services (aPS) for HIV in sub-Saharan is sub-optimal, in part due to poor data quality and limited use of information technology. Consequently, little is known about coverage, scope and quality of HIV aPS. Yet, affordable electronic data tools, software and data transmission infrastructure are now widely accessible in sub-Saharan Africa.

**Methods:**

We designed and implemented a cased-based surveillance system using the HIV testing platform in 18 health facilities in Kenya. The components of this system included an electronic HIV Testing and Counseling (HTC) intake form, data transmission on the Global Systems for Mobile Communication (GSM), and data collection using the Open Data Kit (ODK) platform. We defined rates of new HIV diagnoses, and characterized HIV-infected cases. We also determined the proportion of clients who reported testing for HIV because a) they were notified by a sexual partner b) they were notified by a health provider, or c) they were informed of exposure by another other source. Data collection times were evaluated.

**Results:**

Among 4351 clients, HIV prevalence was 14.2 %, ranging from 4.4–25.4 % across facilities. Regardless of other reasons for testing, only 107 (2.5 %) of all participants reported testing after being notified by a health provider or sexual partner. A similar proportion, 1.8 % (79 of 4351), reported partner notification as the only reason for seeking an HIV test. Among 79 clients who reported HIV partner services as the reason for testing, the majority (78.5 %), were notified by their sexual partners. The majority (52.8 %) of HIV-infected patients initiated their HIV testing, and 57.2 % tested in a Voluntary Counseling and Testing (VCT) site co-located in a health facility. Median time for data capture was 4 min (IQR: 3–15), with a longer duration for HIV-infected participants, and there was no reported data loss.

**Conclusion:**

aPS surveillance using new technologies is feasible, and could be readily expanded into HIV registries in Kenya and other sub-Saharan countries. Partner services are under-utilized in Kenya but further documentation of coverage and implementation gaps for HIV and aPS services is required.

## Background

Assisted Partner Services (aPS) for HIV, the elicitation of sexual history of HIV-infected persons and testing of their sexual partners, is widely accepted as routine public health practice in the United States and Europe [1, 2]. In Africa, aPS has been demonstrated to be feasible, effective and cost-effective, and more studies are ongoing in Kenya to corroborate this evidence [3–6]. aPS could potentially increase HIV testing rates and enhance HIV case finding. However, in Kenya, there are no surveillance systems for case-reporting of HIV-infected index cases and for monitoring the scale up of aPS. Specifically, there is no data on whether HIV Testing and Counseling (HTC) clients are testing due to an exposure from an HIV-infected sexual partner.

Although demographic surveillance systems and nationally representative household surveys are in place, these are expensive, are not timely, and household surveys in particular are not designed to track individuals over time [7]. Furthermore, these surveys are often statistically powered at the national or regional level and advanced analytical techniques are required to generate small area estimates [8, 9]. Routine health information systems (HIS) may be more appropriate for tracking dynamic aPS outcomes including reasons for HIV testing and rates of new diagnoses.

The utility of routine HIS for aPS surveillance, however, is limited by poor data quality, lack of representativeness and the cost involved in collecting and analyzing the data. Also, HIS systems are paper-based, with sub-optimal completeness and delays in reporting. Furthermore, information systems in sub-Saharan Africa do not routinely track patients across the HIV care continuum. Even when client-based data is available, the system for transmission, storage and retrieval is virtually non-existent. Yet, for tracking aPS implementation, real time information systems that leverage on digital platforms, versatile databases and unique personal identifiers are required.

There exists a broad range of robust and flexible devices to manage data, including personal digital assistants (PDAs) and smartphones. These have been applied in public health settings to deliver interventions and measure their impact [10]. Overall, electronic collection of data is cost-effective, is less prone to data loss and is of higher quality than paper-based systems [11–13]. However their use for surveillance is limited, and not adequately described, yet, high quality HIS data should be the norm in sub-Saharan Africa [14–16]. More data are needed on the applicability of electronic collection and transmission of routine HIS data.

In a National Institutes of Health (NIH)-funded implementation science study, we piloted an electronic smart-phone based data collection system using the HIV testing and counselling (HTC) intake form to demonstrate its feasibility and generate baseline data for aPS surveillance. To establish the timeliness, and completeness of electronic collection and transmission of data from the HTC intake form, we explored reasons for HIV testing, characterized HIV-infected patients, and determined the proportion of clients with new HIV diagnoses.

## Methods

### Stakeholder buy-in

At the time of the study, the HIV intake form, a paper based HTC and laboratory register, was the Ministry of Health (MoH) approved tool for collecting and summarizing HIV testing data from health clinics. We engaged the national HTC technical working group (TWG) for approval to insert an additional question on the HIV testing intake form, asking clients: “Why did you test for HIV today?” Clients could indicate one or more reasons. If they chose “Partner Tested Positive” as a reason, clients would further indicate who informed them of their positive partner status: the partner, a health provider or any other method of notification. We then sought and obtained approval from the Health Information Systems (HIS) Department of the Ministry of Health Kenya to utilize the revised form for study purposes. Further to this, we received consent from HIS to convert the intake form electronically and to use smart phones for data collection.

At the health facility level, we discussed the roll-out of the revised tool. Health providers and facility managers advised the study team to implement the electronic form alongside the paper based system so as not to disrupt the existing reporting requirements to the regional and national levels.

### Digitization and data entry

The paper form of the HTC intake form was converted to an electronic format using the ODK platform. ODK Aggregate is an open source application providing a ready-to-deploy server and data repository to provide blank forms to ODK Collect and accept finalized forms from ODK Collect. This software can visualize the collected data using maps and simple graphs, export data, and publish data to external systems. We enhanced the form by developing elaborate skip patterns to the questions and also re-arranged the responses to be more sensitive to the client. Data entry was made using smartphones, with protections against accidental deletion. Once client records were submitted to the server, they were removed from the phone.

### Server, network and user interface

We set up the ODK Aggregate server at the National AIDS/STI Control Programme (NASCOP) with appropriate firewalls. The web connection between smartphones and server was a 256-bit Secure Socket Layer (SSL) Encryption providing an extra layer of protection for system users and data. SSL is the standard security technology that ensures that all data exchanged with a web server remains private and integral. System users were restricted and all users had passwords that met minimum standards of security.

The NASCOP ODK Aggregate servers were built around a virtualized CentOS Linux environment. The CentOS Linux distribution is a stable, predictable, manageable and reproducible platform derived from the sources of Red Hat Enterprise Linux. The CentOS Project is a community-driven free software effort focused around the goal of providing a rich base platform for open source communities to build upon. We implemented a web-based user interface for the server. This interface ran on Apache Tomcat, an open source software implementation of the Java Servlet and Java Server Pages technologies.

### Data transmission

**Fig. 1:**
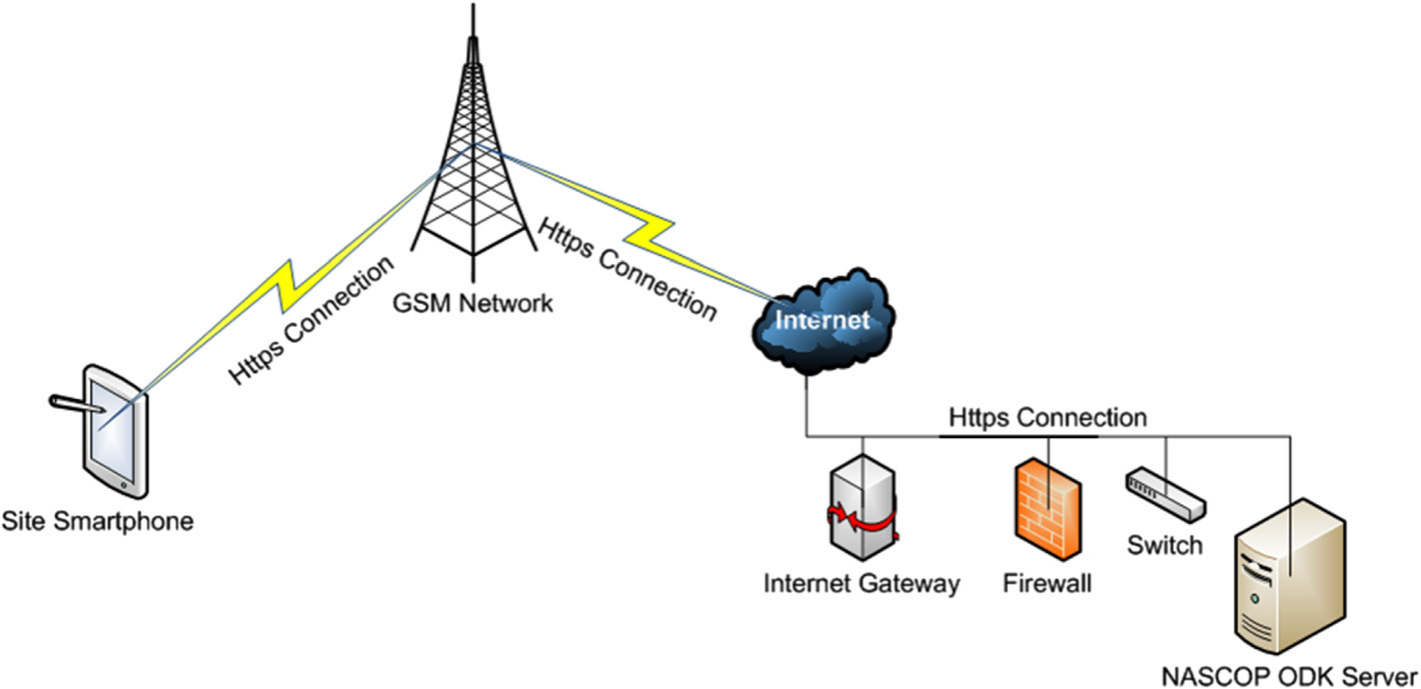
Schematic for electronic data collection, transmission and storage

Network setup for the NASCOP ODK Server was configured on a Gigabit Fiber-optic connection to allow transmission over longer distances with minimal loss and electromagnetic interference. Research assistants uploaded the data at the end of each working day using Global System for Mobile Communication/General Packet Radio Service (GSM/GPRS) network connection (Fig. 1).

### Study settings

The HTC intake form was implemented in all 18 study sites and in tandem with the feasibility and effectiveness aim of the main study [6]. Briefly, the main study was a cluster-randomized trial in which 18 HTC sites were allocated to two groups to evaluate the effectiveness of immediate aPS compared to delayed aPS for HIV. HTC was available either as VCT or provider-initiated (PITC). VCT services were defined as integrated if they were co-located in a health facility. PITC services include testing of pregnant women, patients undergoing medical evaluation and those admitted to inpatient wards. In this study, we enrolled only ambulatory clients who came in to test on their own volition at the designated VCT clinic in each study site. For practical reasons health advisors were free to choose the days and times to collect this data electronically in a way that enabled them to perform other tasks of enrolling and following up for main study. Data was collected in five counties at 18 health facilities as follows: Kiambu (two facilities), Kisumu (five facilities), Muranga (one facility), Nairobi (eight facilities) and Siaya (two facilities)

### Study population

The study population was a purposive sample of clients seeking VCT services at the 18 study sites. All clients aged 18–64 years and presenting at the above study sites for HIV testing were eligible for inclusion.

### Study procedures

After HIV testing, these clients were informed that as standard procedure, their information would be entered into the electronic register in a confidential manner and that they had the right to decline participation. Those who declined participation were not denied any additional service at the VCT clinic. Because this was a public health necessity, we did not make efforts to screen participants for eligibility and neither did we consent them for participation. Upon completion of data entry, the health advisors submitted the data for each client to the Kenya NASCOP server.

### Statistical analysis

Our analysis was based on a convenient sample and consisted of all data collected from the 11^th^ November 2013 and 2^nd^ April 2015. The main outcomes of this study was 1) the completion times of the data capture and 2) the proportion of HIV testing clients who report testing for HIV either because 2a) there were notified by a sexual partner 2b) they were notified by a health provider or 2c) they were informed of exposure by any other source. Because of potential overlapping reasons for HIV testing, the numerator was any positive response to one of the above questions regardless of other reasons for testing. We calculated the proportion of HIV-uninfected participants who self-reported to belong to pre-defined high-risk categories (uncircumcised, ever received money in exchange for sex, engaged in fishing trade, truck driving, with history of having sex with an HIV-infected partner, divorced or separate, in a HIV sero-discordant relationship). We also report the proportion of participants reporting testing for the first time, by HIV status and those with a prior knowledge of HIV status who retest for HIV. We present frequencies, tabulations and bivariate associations based on 4351 observations in five counties.

## Results

### Client characteristics

Out of the 4351 participants, a majority, 2516 (63.3 %), were tested in the integrated VCT clinic. In particular, only 7.1 % tested at the outpatient clinic in the two Kiambu health facilities. The mean age (standard deviation) was 30.1 (9.7), 56.2 % were women and about half were married. The HIV prevalence was 14.2 %, and ranged from 4.4 to 25.4 % across facilities, and among those infected, 17.5 % were not screened for tuberculosis (Table 1). Health facilities in Kiambu and Muranga counties reported a lower prevalence of male circumcision and more than twenty percent of women participants in the Muranga health facility self-reported to be pregnant. Of 720 (16.6 %) who tested as couples, 88 (12.2 %) were sero-discordant. Additionally, 583 (81.0 %) were concordant sero-negative and 45 (6.3 %) were concordant sero-positive. 82 (1.8 %) self-identified as sex workers.

**Table 1:** Socio-demographic, behavioural and biological characteristics of clients, by county, in 18 HTC clinics in Kenya (*N* = 4351 unless otherwise specified)

	Total (*N* = 4351)	Kiambu (*n* = 824)	Kisumu (*n* = 1036)	Muranga (*n* = 113)	Nairobi (*n* = 1964)	Siaya (*n* = 414)
Age(mean,sd)	30.1(9.7)	30.1(9.5)	30.1(11.0)	30.6(10.7)	30.2(8.9)	29.0(10.0)
Sex (Female (*N* (%))	2447(56.2)	494(60.0)	551(53.2)	73(64.6)	1091(55.6)	238(57.5)
Testing Strategy (*N* (%))						
Client-Initiated	2567(59.0)	442(53.6)	470(45.4)	62(54.9)	1363(69.4)	230(55.6)
Provider Initiated	1784(41.0)	382(46.4)	566(54.6)	51(45.1)	601(30.6)	184(44.4)
Facility Testing Venue (*N* (%))						
Integrated VCT clinic	2516(63.3)	733(90.6)	379(47.0)	62(56.9)	1157(60.9)	185(52.1)
General outpatient	1246(31.3)	57(7.1)	375(46.5)	38(34.9)	630(33.2)	146(41.1)
Others	216(5.4)	19(2.3)	52(6.5)	9(8.2)	112(5.9)	24(6.8)
Currently pregnant (*N* (%))^±^	159(6.5)	17(3.4)	22(4.0)	17(23.3)	83(7.6)	20(8.4)
Uncircumcised (*N* (%))^◻^	375(19.7)	10(3.0)	158(32.6)	1(2.5)	127(14.6)	79(44.9)
Married Monogamous (*N* (%))	1994(45.8)	380(46.2)	525(50.7)	49(43.4)	814(41.5)	226(54.6)
HIV-infected (*N* (%))	616(14.2)	55(6.7)	171(16.6)	5(4.4)	306(15.7)	79(19.1)
Tested as couples (*N* (%))^§^	720(16.6)	98(11.9)	192(18.6)	28(24.8)	345(17.7)	57(13.8)
HIV-infected not screened for TB	108(17.5)	0(0.0)	71(41.5)	N/A	24(7.8)	13(16.4)

^±^ *N* = 2447, only women. ^◻^ *N* = 1904, only men. ^§^ *N* = 4332, only couples

### Reasons for testing

**Fig. 2:**
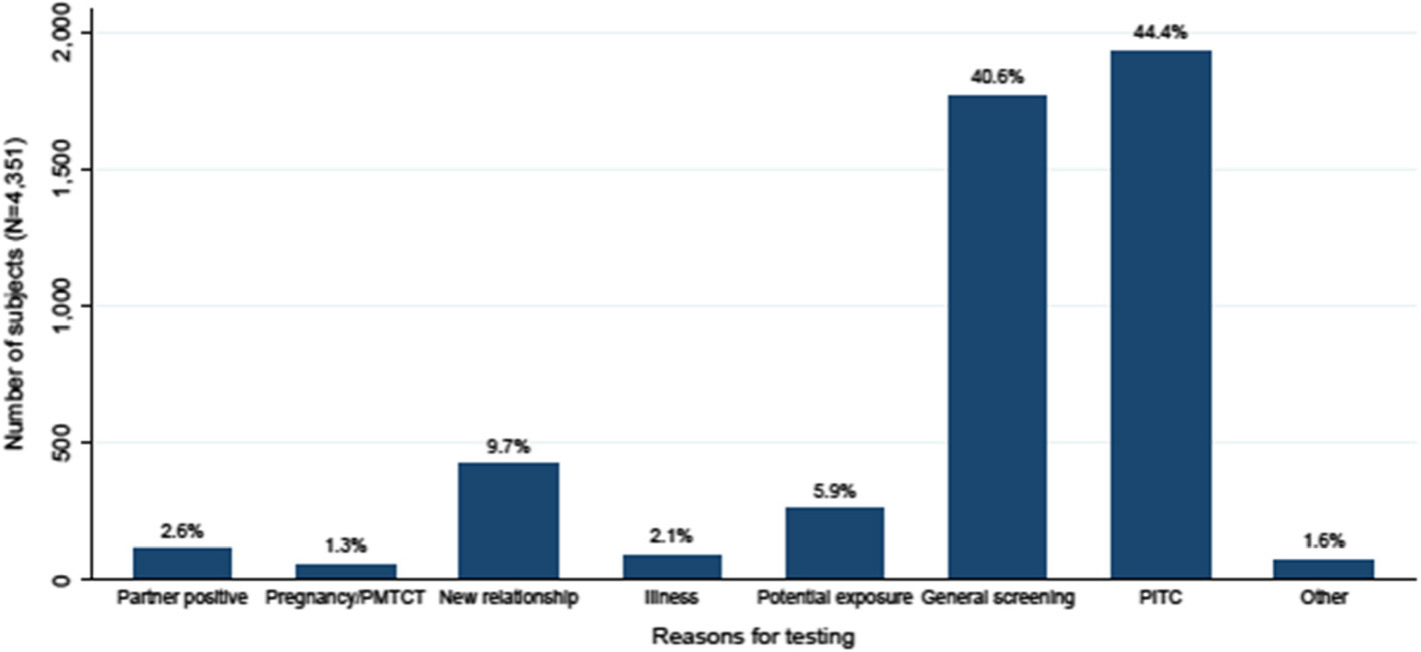
Reasons for HIV Testing for HIV (*N* = 4351)^a^. ^a^Other category includes re- testing by HIV-infected persons (*N* = 36), testing as requirement for marriage, travel or insurance (*N* = 14) and testing for marriage or separation (*N* = 21). These are in addition to other reasons for testing

All 4351 participants gave at least one reason for seeking an HIV test (Fig. 2). A significant proportion reported testing for screening purposes or due to routine offer by health provider (40.6 and 44.4 %, respectively). Only 107 (2.5 %) of persons reported testing as a result of partner notification by a health provider or by an HIV-infected sexual partner, in addition to other reasons. This ranged from 0 to 12.5 % across facilities. Only 79 (1.8 %) respondents reported aPS as the only reasons for testing.

Among the 79 clients who reported partner notification as the only reason for seeking an HIV test, the majority (78.5 %) learnt of their potential exposure through their sexual partners. Only 8.8 % of all partner notifications were reportedly done by the health provider, although with a wide variation across counties (Data not shown). In Muranga and Kiambu, none of the participants tested because a health provider had disclosed to them their potential exposure. However this was approximately 18 % in Kisumu. Other reasons for testing (1.1 % of total) included re-testing by known HIV-positives (*n* = 36), and testing as a requirement for jobs, medical certificates and travel (*n* = 14).

### HIV case detection

Six hundred sixteen (14.2 %) of HTC clients were HIV-infected. The majority, 370 (60.1 %), were women, and the majority, 291(57.2 %) tested for HIV at a VCT co-located in the health facility (Table 2). Approximately half (47.2 %) of these HIV-infected patients tested on their own initiative, and a little over one tenth (15.3 %) tested with their sexual partners. Of the 88 who tested with their partners, 47.9 % reported an HIV-negative. Of the 616 HIV-infected patients, 33 (5.4 %) were fisher folk, 25 (4.1 %) reported engaging in transactional sex, six were truck drivers and one was injecting drugs. Among HIV-infected persons, a third was learning their status for the first time either because they were testing for the first time, or their previous test result was negative. Overall, 720 (16.6 %) tested as couples, with twice the proportion reporting in Muranga testing as such compared to Kiambu.

**Table 2:** Socio-demographic characteristics, and HIV testing behaviors of HIV-infected clients, by county, in 18 HTC clinics in Kenya (*N* = 616 unless otherwise specified)

	Total (*N* = 616)	Kiambu (*N* = 55)	Kisumu (*N* = 171)	Muranga (*N* = 5)	Nairobi(*N* = 306)	Siaya(79)
Age(mean,sd)	32.5(9.1)	32.3(6.9)	32.0(9.5)	38.2(4.8)	33.0(9.2)	31.7(8.9)
Sex (Female (N (%))	370(60.1)	37(67.3)	98(57.3)	5(100.0)	1891(61.8)	41(51.9)
Testing Strategy (N (%))						
Client-Initiated	291(47.2)	23(41.8)	60(35.1)	1(20.0)	165(53.9)	42(53.2)
Provider Initiated	325(52.8)	32(58.2)	111(64.9)	4(80.0)	141(46.1)	37(46.8)
Testing Venue^±^ (N (%))						
Integrated VCT clinic	291(57.2)	36(70.6)	44(38.3)	3(60.0)	175(61.2)	33(63.5)
General outpatient	185(36.4)	11(21.6)	57(49.6)	2(40.0)	98(34.3)	17(32.7)
Others	33(6.4)	4(7.8)	14(12.1)	0(0.0)	13(4.5)	2(3.8)
Testing as Couple	94(15.3)	9(16.4)	22(12.9)	0(0.0)	50(16.3)	13(16.5)
Has HIV-negative partner (N (%))^±^	45(47.9)	7(77.8)	10(45.5)	0(0.0)	25(50.0)	3(23.1)

^±^Among those testing at health facility (*n* = 509). ^±^Among those testing as couples (*n* = 88)

**Fig. 3:**
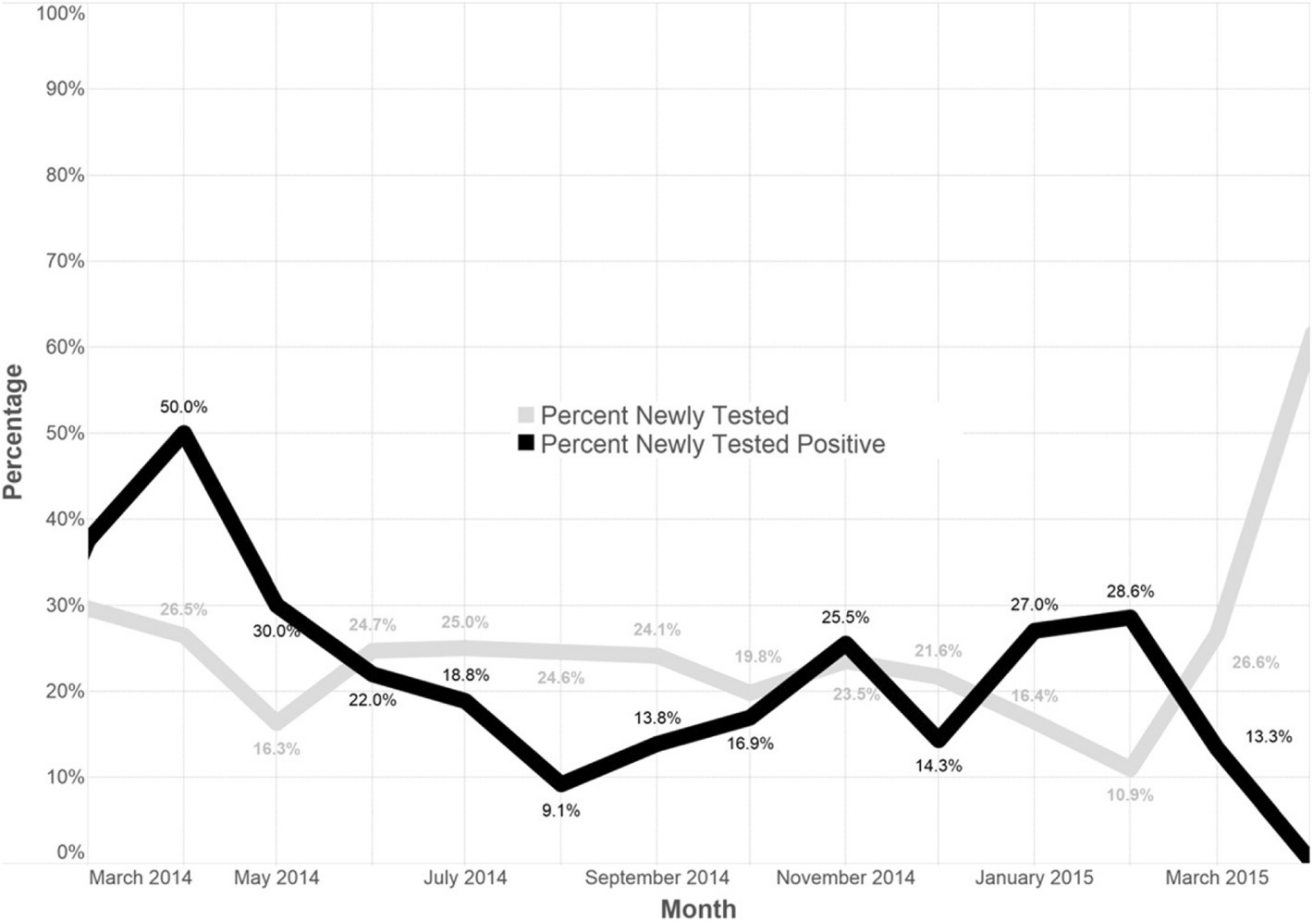
Plot indicating proportion newly tested for HIV and newly diagnosed over 12 months

Over the course of 17 months, the proportion of those newly diagnosed with HIV remained relatively stable although the proportion newly testing appeared to reduce over time (Fig. 3). Across all the sites, the proportion new HTC clients declined from a high of 41.3 % in November 2013 to a low of 10.9 % in February 2015 (data not shown). Although 79.2 % had tested for HIV before, only 16 % of participants were tested as couples.

### Completion times

**Fig. 4:**
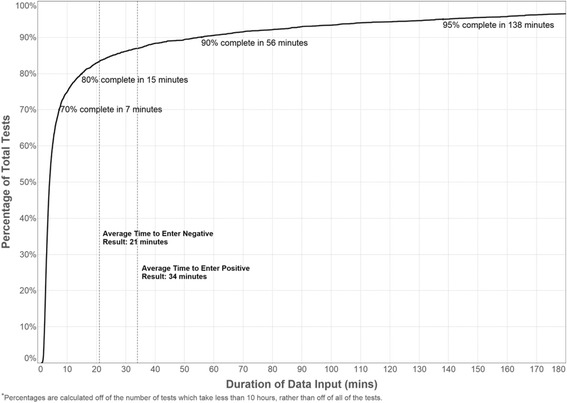
Electronic data capture times, in minutes

Completion times for the HTC intake form are shown in Fig. 4. The median completion time was 4 min (Inter-quartile range [IQR]: 3–15). The median times in minutes (IQR) was 4 (3–11) and 10 (5–42.5) for HIV negative and HIV positive persons respectively. The mean time to enter an HIV negative and an HIV positive result was 21 and 34 min respectively. Approximately 5 % of all test HTC intake forms were not completed even after 2 h. There were no reported data losses during transmission and archiving.

### Missing data

There were no data on sexual history of HIV-infected index cases, including locator information for their sexual partners. Additionally, data on men who have sex with other men (MSM) was not captured in the HTC intake form.

## Discussion

This study showed that the collection, transmission and analysis of routine health information data using mobile smart phones are feasible, with the majority of health advisors collecting the information within ten minutes. This compares well with other studies that have applied smart phones or PDAs to enter routine data [10]. Paper-based forms, may take as much time to fill but it would require daily summaries to be physically transported to district or county health offices where they are manually entered into the web-based national health registry, a process that often takes days [17]. Despite this, a significant time effort is required to establish the infrastructure, enhance network security, develop and pilot the electronic data intake forms. We suggest that an economic evaluation of smart-phone based data collection and transmission should be considered and compared alongside paper-based information systems.

These are the first data from a national surveillance program in Kenya to determine if HTC clients are testing as a result of HIV exposure notification. This will serve as preliminary data for future studies and as baseline for aPS coverage. Although we report a low rate of testing due to notification, it is likely due to low knowledge of HIV status among those infected with HIV. It would be desirable to model what this proportion would have been had a higher proportion of HIV infected persons known their positive status.

The low proportion of high risk population in this study is not surprising. Key populations are often hidden, do not attend health facilities and may be less likely to report their high-risk behaviors to health providers due to stigma [18]. In this report, 31 % of clients tested at the outpatient clinic compared with 44 % in national surveys [19]. This may have limited our ability to detect higher numbers of key populations. Surveillance using health facility HIS therefore may not be suitable for populations at high risk of HIV acquisition. Current surveillance efforts for sex workers, MSMs and People who inject drugs(PWIDS) that include respondent driven sampling, poll booth surveys and venue-based and internet-based sampling techniques should continue [20].

Our findings suggest a high rate of new HIV diagnoses especially in Western Kenya. Whereas this could indicate background incidence of HIV, intensified HIV testing and case finding may have contributed to this phenomenon. The rate of new diagnoses appears to decrease especially in regions with a high HIV testing coverage. This could be an effective way of measuring the success of targeted HIV testing strategies that aim to increase the yield for case detection. It would be desirable to enhance the surveillance tools to further characterize these new HIV diagnoses in terms of their sexual behaviors, partnerships, geospatial location and access to HIV care, and viral suppression. In San Francisco and British Columbia, HIV-cased based systems have been used to estimate HIV outcomes through meticulous documentation of new cases [21, 22]. With sufficient time-series, cased-based HIS provides high-time resolution data and could also be used for measuring impact of interventions [23]. The application of unique personal identifiers and biometrics, combined with an expanded cased-based HIV registry, would be useful in this regard. The support by the HTC TWG for the revised electronic HTC intake form was crucial, but much needs to be done to allay perceptions that this would be parallel system to the HIS that was potentially costly. Beyond demonstrating its cost-effectiveness, smart-phone based systems should demonstrate flexibility and ability to integrate with current HIS platforms.

Future HTC intake forms should include detailed information on sexual partners of HIV-infected cases, including appropriate locator information. Additionally, they should be linked to HIV treatment registries to monitor long term treatment and prevention outcomes of HIV-infected cases and their sexual partners.

Our study had several limitations. Routine facility-based HIS surveillance, may lack representativeness, although this could be improved through increased coverage and weighted estimators to reduce bias [24]. Our study was not nationally-representative and only reflected characteristics of select patients who sought HTC. Estimates for some outcomes in our pilot have been imprecise due to low sample size. A larger sample size and longer time-series would have enabled more robust estimation of new HIV diagnoses. Still, the trends in new HIV tests and diagnoses may have been influenced by disparate patient demographics and the differing clinic staff collecting the data.

## Conclusion

This work helps to establish that electronic aPS surveillance is possible, outlined key implementation gaps for the HIV testing and partner services programs, and laid the foundation for an HIV registry in Kenya.

## Abbreviations

APS, assisted partner services; GPRS, general packet radio service; GSM, Global System for Mobile Communication; HIS, health information system; HTC, HIV testing and counseling; IQR, inter-quartile range; KNH, Kenyatta National Hospital; MoH, Ministry of Health; MSM, men who have sex with other men; NASCOP, National AIDS/STI Control Programme; NIH, National Institutes of Health; ODK, open data kit; PDA, personal digital assistant; PWIDS, people who inject drugs; SSL, secure socket layer; TWG, technical working group; UW, University of Washington; VCT, voluntary counseling and testing

## References

[1] Cates W, Toomey KE, Havlak GR, Bowen GS, Hinman AR (1990). From the CDC. Partner notification and confidentiality of the index patient: its role in preventing HIV. Sexually Transmitted Diseases.

[2] Arthur G, Lowndes CM, Blackham J, Fenton KA (2005). Divergent approaches to partner notification for sexually transmitted infections across the European union. Sexually Transmitted Diseases.

[3] Brown LB, Miller WC, Kamanga G (2011). HIV partner notification is effective and feasible in sub-Saharan Africa: opportunities for HIV treatment and prevention. Journal of Acquired Immune Deficiency Syndromes.

[4] Rutstein SE, Brown LB, Biddle AK (2014). Cost-effectiveness of provider-based HIV partner notification in urban Malawi. Health Policy and Planning.

[5] Henley C, Forgwei G, Welty T (2013). Scale-up and case-finding effectiveness of an HIV partner services program in Cameroon: an innovative HIV prevention intervention for developing countries. Sexually Transmitted Diseases.

[6] Wamuti B (2015). Assisted partner notification services to augment HIV testing and linkage to care in Kenya: study protocol for a cluster randomized trial. Implementation Science.

[7] Waruiru W, Kim AA, Kimanga DO (2014). The kenya AIDS indicator survey 2012: rationale, methods, description of participants, and response rates. Journal of Acquired Immune Deficiency Syndromes.

[8] Srebotnjak T, Mokdad AH, Murray CJ (2010). A novel framework for validating and applying standardized small area measurement strategies. Popul Health Metr.

[9] Hermes K, Poulsen M (2012). Small area estimates of smoking prevalence in London. Testing the effect of input data. Health Place.

[10] Hall CS, Fottrell E, Wilkinson S, Byass P (2014). Assessing the impact of mHealth interventions in low- and middle-income countries--what has been shown to work?. Global Health Action.

[11] Rajput ZA, Mbugua S, Amadi D (2012). Evaluation of an Android-based mHealth system for population surveillance in developing countries. J Am Med Inform Assoc.

[12] Tomlinson M, Solomon W, Singh Y (2009). The use of mobile phones as a data collection tool: a report from a household survey in South Africa. BMC Medical Informatics and Decision Making.

[13] Bernabe-Ortiz A, Curioso WH, Gonzales MA (2008). Handheld computers for self-administered sensitive data collection: a comparative study in Peru. BMC Medical Informatics and Decision Making.

[14] Byass P (2010). The imperfect world of global health estimates. PLoS Medicine.

[15] Chan M, Kazatchkine M, Lob-Levyt J (2010). Meeting the demand for results and accountability: a call for action on health data from eight global health agencies. PLoS Medicine.

[16] Brinkel J, Kramer A, Krumkamp R, May J, Fobil J (2014). Mobile phone-based mHealth approaches for public health surveillance in sub-Saharan Africa: a systematic review. Int J Environ Res Public Health.

[17] Luba PASCOE JL, Jens KAASBØLL, Ismael KOLELENI. Collecting Integrated Disease Surveillance and Response Data through Mobile Phones IST-Africa 2012 Conference Proceedings 2012. Cunningham P and Cunningham M editors. Dar Es Salaam: IIMC International Information Management Corporation; 2012. ISBN 978-1-905824-34-2.

[18] Beyrer C, Baral SD, van Griensven F (2012). Global epidemiology of HIV infection in men who have sex with men. Lancet.

[19] Ng’ang’a A, Waruiru W, Ngare C (2014). The status of HIV testing and counseling in Kenya: results from a nationally representative population-based survey. Journal of Acquired Immune Deficiency Syndromes.

[20] Shaghaghi A, Bhopal RS, Sheikh A (2011). Approaches to Recruiting ‘Hard-To-Reach’ Populations into Re-search: A Review of the Literature. Health Promot Perspect.

[21] Montaner JS, Lima VD, Barrios R (2010). Association of highly active antiretroviral therapy coverage, population viral load, and yearly new HIV diagnoses in British Columbia, Canada: a population-based study. Lancet.

[22] Ahrens K, Kent CK, Kohn RP (2007). HIV partner notification outcomes for HIV-infected patients by duration of infection, San Francisco, 2004 to 2006. Journal of Acquired Immune Deficiency Syndromes.

[23] Wagenaar BH, Sherr K, Fernandes Q, Wagenaar AC. Using routine health information systems for well-designed health evaluations in low- and middle-income countries. Health Policy Plan. 2016;31(1):129-35. doi:10.1093/heapol/czv029. Epub 2015 Apr 16. 10.1093/heapol/czv029 PMC4751224 25887561

[24] Souty C, Turbelin C, Blanchon T, Hanslik T, Le Strat Y, Boelle PY (2014). Improving disease incidence estimates in primary care surveillance systems. Popul Health Metr.

